# Nasal Delivery of Acute Medications for Migraine: The Upper Versus Lower Nasal Space

**DOI:** 10.3390/jcm10112468

**Published:** 2021-06-02

**Authors:** Vincent Martin, John Hoekman, Sheena K. Aurora, Stephen B. Shrewsbury

**Affiliations:** 1 University of Cincinnati Headache and Facial Pain Center, Cincinnati, OH 45219, USA; martinvt@ucmail.uc.edu; 2 Research & Development, Impel NeuroPharma, Seattle, WA 98119, USA; jhoekman@impelnp.com; 3 Medical Affairs, Impel NeuroPharma, Seattle, WA 98119, USA; saurora@impelnp.com; 4 Clinical Development, Impel NeuroPharma, Seattle, WA 98119, USA

**Keywords:** migraine, headache, acute treatment, therapy, nasal delivery, drug delivery, bioavailability, upper nasal space, olfactory region

## Abstract

The acute treatment of migraine requires effective drugs that are well tolerated and provide rapid and consistent pain relief. Oral tablets are the most commonly used acute treatment for migraine; however, their effectiveness is limited by the rate of gastrointestinal (GI) tract absorption and first-pass hepatic metabolism, and they may not be ideal for patients experiencing GI motility issues. Nasal delivery is an attractive alternative route as it may circumvent GI tract absorption, avoid first-pass metabolism in the liver, and potentially reduce the frequency of GI adverse events. The large surface area and high vascularity within the nose may permit rapid absorption of therapeutics into the systemic circulation, allowing for rapid onset of action. However, the site of drug deposition (upper versus lower nasal cavity) may influence drug pharmacokinetics. Most approved nasal migraine therapies target the lower nasal space where the epithelium is less permeable, and they may be quickly cleared away due to increased ciliary function or dripping from the nose or swallowing, resulting in variable absorption and limited bioavailability. Together with its abundant vascularization, relative mucosal thickness stability, and low clearance rates, the upper nasal space harnesses the benefits of nasal delivery to potentially maximize drug efficacy.

## 1. Introduction

Migraine is a debilitating condition, representing the second leading cause of disability globally [1,2,3,4]. In a recent epidemiological study in the United States (US), 19.2% of individuals have self-reported a migraine, with 15.8% reporting at least one monthly headache day over a 3-month period. Of the 18,353 respondents who met criteria for migraine, migraine disproportionately affected women versus men. Of the 15,133 (women; *n* = 11,049, men; *n* = 4084) reporting at least one migraine headache day per month, the ratio of women to men with migraine was 3:1 [5]. Overall, 18% of American women and 6% of men experience migraine headaches [6].

It is estimated that more than 90% of individuals use medication for the acute treatment of their migraine headaches [7,8]. However, approximately 36% of those who use medication for their headaches discontinue treatment, and lack of treatment efficacy ranks among the top reasons for suspending treatment [8,9]. While numerous administration routes for acute therapy for migraine exist, oral tablets are the most commonly prescribed for patients, totaling over 90% of therapies prescribed [5]. Oral drugs offer unique advantages, including portability and ease of administration [10]. However, bioavailability, or the rate and extent to which a drug reaches the systemic circulation, becomes a challenge with oral administration because oral drugs must first pass through the stomach and into the small intestine of the gastrointestinal (GI) system before they can be absorbed [11,12]. Bioavailability is subject to variations in intestinal absorption, and in addition, even well-absorbed drugs may then undergo high rates of hepatic first-pass metabolism, which may make them pharmacologically inactive [10,11,12,13]. Additionally, disparities in gut motility and metabolism among individuals, and even within an individual on different occasions, may affect bioavailability [12,14]. A drug that is rapidly absorbed in the GI system of a healthy individual may stall in the stomach of some migraine patients. Most pharmacokinetic studies are conducted in healthy volunteers, with the exception of those identified by the Food and Drug Administration (FDA) that should be conducted in a defined patient population [12,15]. While it would be ideal to conduct pharmacokinetic studies of oral migraine drugs in migraine patients during an attack, it is not practical; therefore, other methods to understand the impact of gut motility in the patient population must be employed in order to effectively assess drug absorption and bioavailability. Finally, oral medications may not be ideal for migraine patients experiencing gastroparesis, as delayed absorption may allow migraine symptoms to worsen. Migraine commonly accompanied by nausea may lead to reluctance to take oral treatment, and vomiting may lead to ingested drugs being lost in vomitus and uncertainty about whether to re-dose. [11,16,17,18,19]

Alternative routes of administration for the acute treatment of migraine include injection (subcutaneous (SC), intramuscular (IM), or intravenous (IV)), transdermal, and inhalation (nasal and pulmonary) [17,19,20]. Injection and inhalation (both pulmonary and nasal) offer rapid relief (<15 min) from migraine; although IV injection may be faster and more consistent than SC or IM injection, it requires staff to administer [17,19,21]. While IV injection completely bypasses gastric stasis issues and hepatic first-pass metabolism, some drugs may worsen nausea and vomiting or other concentration-dependent adverse events (AEs) due to a rapid increase in plasma and brain concentrations. Injections may also represent a problem for those patients who are averse to needles [19]. Advantages of inhaled delivery include at-home administration, non-invasiveness, and easy self-administration as well as avoidance of drug degradation in the GI tract and first-pass metabolism (similar to injection), which allows for enhanced bioavailability and reduction of systemic side effects without the use of a needle [22,23].

Migraine and disorders of the nose can be comorbid conditions, which suggests a possible underlying pathophysiological relationship [24]. Rhinitis is a comorbidity of migraine, and epidemiological studies have shown that a relationship between rhinitis and migraine exists [25,26]. A 2008 questionnaire study demonstrated that the frequency of migraine attacks is significantly increased in patients with rhinitis and that rhinitis is associated with increased headache disability [26]. Another study demonstrated that migraine was significantly more prevalent in patients with rhinitis (34%; *p* < 0.001) and that the odds of experiencing a migraine attack were 14.3 times higher in patients with rhinitis [25]. Additionally, unilateral cranial autonomic symptoms (UASs), which include nasal congestion and rhinorrhea, can occur during a migraine attack. A 2006 study revealed that migraine patients with UASs experienced longer migraine duration, increased head pain severity, and more frequent allodynia and photophobia compared to migraine patients without UASs [27]. Olfactory triggers are common in those with migraine, occurring in 90% of migraine patients in a 2016 study. In those patients who experienced odor-triggered migraine attacks, osmophobia was reported by 95% of patients. Additionally, reduced olfactory acuity was reported by patients who experienced osmophobia and odor-triggered headaches [28]. Such evidence underpins a connection between the nose and migraine, and thus a nasal treatment may make more logical sense than previously appreciated.

Nasal delivery has promised to be an effective alternative route of administration for over 25 years, yet despite many effective migraine drugs being formulated for nasal administration, they have failed to generate the consistent or convincing efficacy data for all patients with all migraine attacks and most have not been commercially successful [23,29,30]. This review will discuss the shortcomings of approved nasally delivered therapies and the current attempts to overcome the issues associated with traditional nasal delivery of acute medications for migraine. The nose is a complex organ and the need to deliver efficacious medication to the appropriate anatomical region of the nose may have been underestimated.

## 2. Nasal Delivery: All Parts of the Nose Are Not the Same

**Figure 1:**
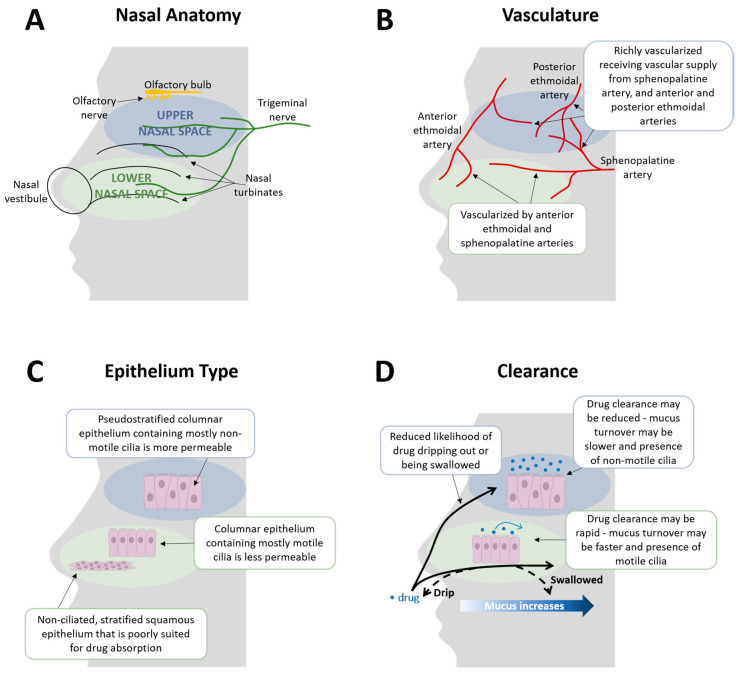
Anatomy, vasculature, epithelia, and clearance in the upper versus lower nasal space. (**A**) The nose is divided into an upper and a lower nasal space [33,34]. The lower nasal space is located just posterior to the nasal openings and includes the vestibule and the nasal turbinates [33]. It is innervated by the trigeminal nerve [11,35]. The upper nasal space contains the upper portion of the superior turbinate, the inferior surface of the ethmoid bone, and the olfactory region [33,35]. It is innervated by both the olfactory and trigeminal nerves [11]. (**B**) The vascular supply in the lower nasal space derives from branches of the ophthalmic and maxillary arteries, specifically the anterior ethmoidal artery and the sphenopalatine artery [11,42]. The upper nasal space is abundantly vascularized, receiving vascular supply from the sphenopalatine artery and the anterior and posterior ethmoidal arteries [43]. (**C**) The lower nasal space contains mostly non-ciliated, stratified squamous epithelium in the vestibular region, which is poorly suited for drug absorption, and ciliated columnar cells predominate in its respiratory region [35,37]. Pseudostratified columnar epithelium containing mostly non-motile cilia is present in the olfactory epithelium of the upper nasal space [37,44]. The olfactory epithelium is more permeable than the respiratory epithelium and may allow for better drug absorption [23,45]. (**D**) Within the nasal cavity, there is the presence of mucus which a drug must pass through, and mucus increases in an anterior to posterior manner [46,47,48]. Mucus in the olfactory region is produced and secreted by Bowman’s glands, and from both seromucous glands and goblet cells in the respiratory mucosa [37]. A rodent study demonstrated that mucus in the olfactory mucosa moves slowly, with a turnover time of several days, while mucus in the respiratory region turns over rapidly, with an estimated turnover time of approximately 10 min [46,49]. Potentially slower mucus turnover, and decreased presence of motile cilia in the upper nasal space, may result in reduced mucociliary clearance from the upper nasal region [37,44]. Additionally, drug delivery to the upper nasal space may have a reduced likelihood of dripping out or being swallowed [23,34,37]. Compared to the lower nasal space, drug delivery to the upper nasal space may provide greater, more consistent drug absorption, and thus may reduce response variability and provide greater, rapid relief [34,50,51]. Note: Images are not drawn to scale.

The upper respiratory tract includes the nasal cavities and passages, pharynx, tonsils, and larynx, while the trachea and lungs make up the lower respiratory tract [31,32]. The upper respiratory tract serves to filter, humidify, and warm the air that is delivered to the lower respiratory tract [32]. The nose can be divided into an upper and a lower nasal space (Figure 1) [33,34]. The lower nasal space is located more anteriorly, just posterior to the nasal openings, and includes structures such as the vestibule and the nasal turbinates [33]. The upper nasal space lies just beyond the lower nasal space and contains the upper portion of the superior turbinate, the inferior surface of the ethmoid bone, and the olfactory region [33,34,35]. The upper and lower nasal spaces differ in epithelium type, ciliary function, vascular supply, but share innervation by the trigeminal nerve [11,36,37]. The trigeminal nerve innervates both the lower and upper nasal spaces and has been considered a common denominator in headache pathophysiology as it also supplies sensory innervation to other parts of the head and face [36,37,38]. It has been termed a “central hub” in the trigeminovascular pathway transmission because it ultimately signals to the cortex through hypothalamic activation [38]. The trigeminal nerve receives and processes nociceptive and sensory signals and innervates intracranial vasculature. Its sensory receptors cover meningeal perivascular nerves of pial and dural blood vessels [38,39]. Thalamic trigeminovascular neurons contribute to migraine pain and mediate many migraine-associated symptoms, such as phonophobia, photophobia, allodynia, and osmophobia [39,40,41].

### 2.1. Lower Nasal Space

Within the lower nasal space lies the most anterior vestibular region immediately posterior to the nostril openings. This region contains nasal hairs that filter inhaled particles and non-ciliated, stratified squamous epithelium. It encompasses a relatively small total surface area of 0.6 cm^2^, which may be a possible reason for minimal drug absorption in this area [11,33,35,37]. The respiratory region lies just posterior to the vestibular region and has a large mucosal surface area of approximately 130 cm^2^, covering the lateral walls of the nasal cavity and the three projecting nasal turbinates (Figure 1) [35,52]. This region contains ciliated respiratory epithelium, with nearly 80% of the cells within the respiratory mucosa containing motile cilia [11,37]. Mucus production in this area comes from seromucous glands and goblet cells [37,44] The presence of mucus increases in an anterior to posterior gradient [46,47,48]. It turns over rapidly, with an estimated turnover time of approximately 10 min as assessed in the rodent [46,49]. The respiratory region is vascularized by branches of the ophthalmic and maxillary arteries supplying the mucosal membranes of this region, and its neural innervation comes from the trigeminal nerve [11,35]. While its large surface area and high vascularity make the respiratory region within the lower nasal space a promising location for drug delivery, absorption across the respiratory mucosa of the vestibule, lower nasal space, and associated middle and lower turbinates may be variable. Underlying mucosal edema from allergic or infectious disease may lead to inconsistent response, or mucus trapping and then clearing the drug via mucociliary clearance may slow absorption [34,35,37,44,53]. Most nasal sprays have been developed to deliver to the lower nasal space [11,33]. However, limited and inconsistent systemic absorption has been reported with traditional nasal sprays, which were specifically developed to treat local nasal and upper airway diseases in which systemic absorption is neither required nor desired (e.g., when using corticosteroids or decongestants for allergic or viral rhinitis) [23,33]. Drugs delivered to the lower nasal space may be quickly swept away by mucociliary clearance or drained away due to gravity from the nasal cavity and driven toward the nasopharynx, where they are swallowed or expectorated, resulting in variable absorption [23,34,37,44]. While several nasal therapies for the treatment of migraine exist, traditional nasal spray devices deposit less than 5% of the active drug into the upper nasal space and have been developed to deliver to the lower nasal space, where their rapid clearance results in limited bioavailability and inconsistent clinical results [11,23,37].

### 2.2. Upper Nasal Space

Beyond the respiratory region in the superior aspect of the nasal cavity lies the upper nasal space, which contains the olfactory mucosa [34,35]. The olfactory region consists of a pseudostratified columnar epithelium covering the septum, the upper portion of the superior turbinate, the lateral surface of the posterosuperior portions of both nasal cavities, and the inferior surface of the ethmoid bone’s cribriform plate [35,43,54]. Mucus is produced and secreted by Bowman’s glands in this region, and moves slowly, with a turnover time of several days as assessed in the rodent [37,44,46,49]. The olfactory region is abundantly vascularized, receiving vascular supply from the sphenopalatine artery and anterior and posterior ethmoidal arteries [43]. Neural innervation to this area is supplied by both the olfactory and trigeminal nerves [11,36,37]. Unlike the respiratory mucosa in the lower nasal space where nearly all cells are covered with motile cilia, non-motile cilia are found in the olfactory mucosa in the upper nasal region [37]. However, gravitational forces and small islets of respiratory mucosa containing motile cilia in this region do contribute to mucociliary clearance [37,44]. The decreased presence of motile cilia in the upper nasal space may result in reduced mucociliary clearance in this cavity. Therefore, delivery of a therapeutic to this space may result in increased absorption, which otherwise would not have occurred if the drug were quickly cleared away [23,37,55,56,57]. Additionally, the olfactory epithelium is more permeable than the respiratory epithelium [45]. A rich network of blood and lymphatic vessels present in the underlying submucosa of the olfactory region may enhance drug absorption by draining substances, that are not absorbed into the bloodstream, into the lymphatic system for systemic absorption [22,44,50]. Compared to the lower nasal space, delivery of drugs to the upper nasal space has the potential to provide greater, more consistent drug absorption, and thus may reduce response variability and provide reliable relief in a non-invasive manner [23,34,50,51].

### 2.3. Additional Factors That May Impact Nasal Drug Delivery

Other factors that can influence the efficiency of nasal delivery besides the location of drug deposition within the nose include physiochemical properties of the active pharmaceutical ingredient (API; e.g., molecular size, lipophilicity/polarity/ionic charge, enzymatic degradation in the nose), as well as properties of its formulation (e.g., dry powder, liquid, concentration, pH, osmolarity, viscosity) [58]. Just as device factors (e.g., spray pattern, plume geometry, dose volume, droplet size distribution, velocity) can influence where in the nose the drug is deposited, formulation strategies (e.g., enzyme inhibitors, permeation enhancers, particulate design) can also influence uptake across the nasal mucosa [58,59].

## 3. Overview of Nasal Products That Are in Development or Approved, Including Efficacy, Safety, and Bioavailability

There are currently several nasal products that are approved for the acute treatment of migraine. While nasal powder formulations exist, the nasal drug market is currently dominated by liquid formulations [60]. They are summarized below and in Table 1.

### 3.1. Approved Products

#### 3.1.1. IMITREX^®^

IMITREX (GlaxoSmithKline, Research Triangle Park, NC, USA) was approved in the US in 1997 as a nasal spray. It delivers sumatriptan, a serotonin receptor (5-HT_1B/1D_) agonist, via a standard nasal spray and is indicated for the acute treatment of migraine with or without aura [29,60,61]. The recommended adult dose is 5, 10, or 20 mg, with 5 mg and 20 mg doses administered in a single spray and the 10 mg dose in two sprays. Each 100 µL spray of IMITREX contains 5 or 20 mg sumatriptan in an aqueous buffered solution containing monobasic potassium phosphate National Formulary (NF), anhydrous dibasic sodium phosphate United States Pharmacopeia (USP), sulfuric acid NF, sodium hydroxide NF, and purified water USP. The solution’s pH is approximately 5.5 with an osmolality of 372 or 742 mOsmol for the 5 and 20 mg doses, respectively. The mean bioavailability following nasal administration was 17% relative to SC sumatriptan injection [29]. The maximum observed plasma concentration (C_max_) was 69.5 ng/mL and 12.9 ng/mL following SC and nasal administration of sumatriptan, respectively, and the rate of absorption (measured as area under the serum concentration–time curve from zero to t_max_, AUC_tmax_) was 9.0 and 7.4 h*ng/mL for SC and nasal sumatriptan administration, respectively [79]. A total of eight randomized, double-blind, placebo-controlled clinical trials were performed to assess safety, efficacy, and tolerability of IMITREX nasal spray, of which five used the recommended dosing regimen and marketed formulation and are included in the United States Prescribing Information (USPI) [29]. In all trials, doses of 10 and 20 mg were compared with placebo in the treatment of one to three migraine attacks, and in two trials, a 5 mg dose was also evaluated against placebo. Trial 5 was a multi-attack study, but only data for the first attack were reported in the USPI [29,80]. These studies demonstrated that a significantly greater percentage of patients achieved headache relief (defined as no or mild pain) at 2 h post-treatment with 20 mg IMITREX nasal spray compared to those receiving the placebo in Trial 1 (64% versus 25%, *p* < 0.05), Trial 2 (55% versus 25%, *p* < 0.05), Trial 3 (63% versus 35%, *p* < 0.05), Trial 4 (62% versus 29%, *p* < 0.05), and Trial 5 (60% versus 36%, *p* < 0.05) [29]. Similar observations extended to patients who received 10 mg IMITREX nasal spray in Trial 1 (46% versus 25%, *p* < 0.05), Trial 2 (44% versus 25%, *p* < 0.05), Trial 3 (54% versus 35%, *p* < 0.05), Trial 4 (43% versus 29%, *p* < 0.05), and Trial 5 (53% versus 36%, *p* < 0.05). The most commonly reported AEs (≥1% of patients and greater than placebo) in those who received 20, 10, or 5 mg IMITREX nasal spray were similar although they occurred at different frequencies. Burning sensation was experienced in 1.4, 0.6, and 0.4% of patients who received 20, 10, and 5 mg, respectively. Disorder/discomfort of nasal cavity/sinuses was reported by 3.8, 2.5, and 2.8% in those who received 20, 10, and 5 mg, respectively. Throat discomfort was reported by 2.4, 1.8, and 0.8% in those who received 20, 10, and 5 mg, respectively. Nausea and/or vomiting was reported by 13.5, 11.0, and 12.2% of those who received 20, 10, and 5 mg, respectively. Bad/unusual taste was reported by 24.5, 19.3, and 13.5% in those who received 20, 10, and 5 mg, respectively. Finally, dizziness/vertigo was experienced by 1.4, 1.7, and 1.0% of patients who received 20, 10, and 5 mg, respectively [29]. No consistency of response data or long-term efficacy, safety, and tolerability data were found in published literature.

#### 3.1.2. MIGRANAL^®^

MIGRANAL (Bausch Health Companies Inc. or its affiliates, Bridgewater, NJ, USA) received US approval in 1997 and is a dihydroergotamine (DHE) mesylate nasal spray indicated for the acute treatment of migraine with or without aura [23,62]. It utilizes a traditional nasal delivery system that targets DHE mesylate to the lower nasal space [23,51]. In addition to DHE mesylate, each dose contains anhydrous caffeine, anhydrous dextrose, carbon dioxide, and purified water and its pH is approximately 4.4–5.4 in solution [62,81]. MIGRANAL acts by binding to serotonergic, dopaminergic, and adrenergic receptors. The recommended dosing is a single 0.5 mg/mL spray administered in each nostril, followed by one additional spray in each nostril 15 min later, for a total of 2 mg MIGRANAL delivered in four sprays. The mean bioavailability following nasal administration was 32% relative to injectable administration [62]. Several studies assessed the pharmacokinetics of MIGRANAL, with results demonstrating a t_max_ of 45 min and a dose–bioavailability relationship based on C_max_ and AUC values of plasma DHE shown to be linear in the 0 to 4 mg range [81]. Four randomized, double-blind, placebo-controlled, single-dose clinical trials were carried out in the US and are described in the USPI [62]. A significantly greater percentage of patients achieved headache relief (defined as no or mild pain) at 4 h with 2 mg MIGRANAL compared to placebo in three of the four studies (Study 1, 70% versus 28%, *p* < 0.001; Study 2, 56% versus 35%, *p* < 0.01; Study 3, 48% versus 22%, *p* < 0.01) [62,63]. AEs reported by ≥1% of MIGRANAL-treated patients and that occurred more frequently than in the placebo group in the controlled studies reported in the USPI included rhinitis (26%), nausea (10%), altered sense of taste (8%), application site reactions (6%), dizziness (4%), and vomiting (4%). It is important to note that the prescribing information for MIGRANAL includes a black box label reporting that serious and/or life-threatening peripheral ischemia has been associated with coadministration of DHE and CYP (cytochrome P450) 3A4 inhibitors [62]. No consistency of response data or long-term efficacy, safety, and tolerability data were found in published literature.

#### 3.1.3. ZOMIG^®^

ZOMIG (Amneal Pharmaceuticals, Bridgewater, NJ, USA) was approved in 2003 in the US [64,65]. It is a zolmitriptan-containing nasal spray indicated for the acute treatment of migraine with or without aura that has been shown to deliver zolmitriptan to the nasopharynx and lower nasal space [33,64,82]. It is a serotonin receptor agonist with a recommended starting dose of 2.5 mg; however, 5 mg can be used if headache relief is not achieved with 2.5 mg. Each 100 µL dose is supplied in an aqueous buffered solution containing anhydrous citric acid, disodium phosphate dodecahydrate USP, and purified water USP in addition to 2.5 or 5 mg of zolmitriptan buffered to pH 5.0. The solution is hypertonic and its osmolarity is 360 to 420 and 420 to 470 mOsmol for the 2.5 and 5 mg dose, respectively. The mean bioavailability following nasal administration of ZOMIG is 102% relative to an oral tablet form [64]. The mean bioavailability of oral zolmitriptan is 49% relative to IV zolmitriptan [83]. A single randomized, outpatient, double-blind, dose-ranging, multi-attack placebo-controlled trial was performed in adults [66], and a randomized, double-blind, dose-ranging, single-attack placebo-controlled trial with a single-blind run-in period was performed in adolescents [67] in order to establish efficacy. A significantly greater percentage of patients achieved headache relief (defined as no or mild pain) at 2 h with 2.5 mg ZOMIG compared to placebo in adults (55% versus 31%, *p* < 0.001) and in adolescents (53% versus 39%, *p* < 0.05). Similar results were observed with 5 mg ZOMIG compared to placebo in adults (69% versus 31%, *p* < 0.001) and adolescents (51% versus 39%, *p* < 0.05) [64,66,67]. The adult study revealed that the 2-hour headache response to ZOMIG was maintained consistently across the treatment of multiple attacks [66]. The most commonly reported AEs that occurred in ≥2% of adult or pediatric patients taking either 2.5 or 5 mg ZOMIG in controlled studies were unusual taste (5 mg ZOMIG, 21%; 2.5 mg ZOMIG, 17%), paresthesia (5 mg ZOMIG, 10%; 2.5 mg ZOMIG, 5%), hyperesthesia (5 mg ZOMIG, 5%; 2.5 mg ZOMIG, 1%), and somnolence (5 mg ZOMIG, 4%; 2.5 mg ZOMIG, 1%) in adults and unusual taste (5 mg ZOMIG, 10%; 2.5 mg ZOMIG, 6%) in adolescents [64]. Long-term studies revealed that 5 mg ZOMIG provided consistent tolerability and efficacy [68,84]. A randomized, double-blind, parallel-group, multicenter study designed as a two-phase, crossover trial assessed long-term tolerability, efficacy, and consistency of response of ZOMIG over 1 year. Consistency of response was reported in 56.9% of patients taking 5 mg ZOMIG who achieved a 2-hour headache response in >75% of attacks over the course of 1 year [84]. An open-label, noncomparative, multicenter, Phase 3 study assessed the long-term safety and tolerability of 5 mg ZOMIG in treating multiple migraine attacks over 1 year in migraine patients mostly naïve to triptan nasal sprays. A total of 538 patients treated 20,717 migraine attacks. The number of migraine attacks treated per patient was large (mean of 38.5 attacks per patient, with 79.7% of patients treating ≥10 attacks) and the majority (58.6%) demonstrated repeated and extended use of ZOMIG, treating a mean of ≥2 attacks per 30-day period for ≥6 months. In this long-term safety and tolerability study, the only AEs to occur in ≥4% of attacks were unusual taste (19%) and paresthesia (6.8%) [68].

#### 3.1.4. ONZETRA^®^ Xsail^®^

ONZETRA Xsail (Currax Pharmaceuticals LLC, Morristown, NJ, USA) received US approval in 2016 [69]. It is a sumatriptan nasal powder contained in a disposable, single-use nosepiece containing 11 mg sumatriptan base in a clear hypromellose capsule attached to a reusable delivery device body containing a mouthpiece and piercing mechanism. It is delivered into the nostril by blowing through the mouthpiece while the nosepiece is inserted into one nostril. The recommended dosing is achieved by delivering the contents of one 11 mg nosepiece to each nostril for a total of 22 mg [70]. Breath-powered nasal delivery of ONZETRA Xsail may allow for delivery of some sumatriptan to the upper nasal space [71]. Following nasal administration, the mean bioavailability of sumatriptan was 19% relative to SC injection [70]. A study comparing the pharmacokinetics of 20 mg sumatriptan nasal spray (IMITREX nasal spray), 22 mg sumatriptan nasal powder (ONZETRA Xsail), 100 mg sumatriptan oral tablet (IMITREX tablet), and 6 mg sumatriptan injection (IMITREX injection) found that administration of sumatriptan powder using the breath-powered technology of ONZETRA Xsail resulted in 27% higher C_max_ (20.8 vs. 16.4 ng/mL) and a 75% higher early exposure (AUC_0-15 min,_ 2.1 vs. 1.2 ng*hour/mL) relative to the IMITREX nasal spray despite utilizing a 20% lower dose. However, relative to both the IMITREX tablet and injection, the peak (C_max_, 20.8, 70.2, 111.6 ng/mL for ONZETRA Xsail, IMITREX tablet, and IMITREX injection, respectively) and overall exposure (AUC_0-inf_, 64.9, 308.8, 128.2 ng*hour/mL for ONZETRA Xsail, IMITREX tablet, and IMITREX injection, respectively) following ONZETRA Xsail were lower [85]. One multicenter, randomized, double-blind, placebo-controlled, single-attack study is described in the USPI, which reported that a significantly greater percentage of patients achieved headache relief (defined as no or mild pain) at 2 h with 22 mg ONZETRA Xsail compared to placebo (68% versus 45%, *p* < 0.05) [70,72]. Additional medications were allowed as rescue therapy 2 h after initial treatment. No headache symptoms (pain freedom and no nausea, no photophobia, and no phonophobia) were reported by 34% of patients who received 22 mg ONZETRA Xsail compared to 17% of patients who received placebo (*p* < 0.05) at 2 h post-treatment. The most common AEs reported by ≥2% of patients were abnormal taste (20%), nasal discomfort (11%), rhinorrhea (5%), and rhinitis (2%) [70]. Consistency of response across three migraine attacks with 22 mg ONZETRA Xsail was compared to 100 mg oral sumatriptan in a randomized, multicenter, double-dummy, crossover, multi-attack, comparative efficacy study with two 12-week double-blind periods. Results revealed that a significantly greater percentage of patients had consistent pain relief and pain freedom with ONZETRA Xsail across multiple attacks at 30 min post-dose (*p* < 0.05) [73]. Another analysis of consistency from the same study mentioned above used a novel analytic technique and revealed a greater within-person consistency across multiple migraine headaches from 45 to 120 min post-dose with ONZETRA Xsail compared to oral sumatriptan [86]. Although it has been suggested that ONZETRA Xsail may deliver sumatriptan to the upper nasal space [71], no upper nasal safety assessment of olfactory mucosa structure and function by endoscopic evaluation or olfactory function testing, respectively, has been performed. No long-term efficacy, safety, or tolerability data were found in published literature.

#### 3.1.5. TOSYMRA™

TOSYMRA (Upsher-Smith Laboratories, Maple Grove, MN, USA) was approved in 2019 in the US and is a nasal spray containing sumatriptan and a permeation-enhancing excipient (0.2% 1-O-*n*-Dodecyl-β-D-maltopyranoside (DDM, Intraveil^®^)) indicated for the acute treatment of migraine with or without aura [74,75]. Its site of drug deposition within the nasal cavity has not been explicitly stated in the literature. However, inclusion of the permeation-enhancing excipient, DDM, can enhance absorption and bioavailability of drugs delivered intranasally [76,87,88]. In addition to sumatriptan and DDM, each 100 µL dose is supplied in an aqueous buffered solution containing citric acid monohydrate, potassium phosphate monobasic, sodium chloride, and anhydrous sodium phosphate dibasic. Its pH is approximately 5.0–6.0 and its osmolality is between 270 and 330 mOsmol [74]. The recommended dose for TOSYMRA is 10 mg administered in a single spray in one nostril, and the mean bioavailability following nasal administration of TOSYMRA 10 mg is 87.7% relative to a 4 mg SC injection and 58.4% relative to 6 mg SC injection [74,77]. C_max_ was 51.8, 49.1, and 72.8 ng/mL for TOSYMRA 10 mg, 4 mg SC injection, and 6 mg SC injection, respectively, and AUC_0–∞_ was 60.7, 69.2, and 103.8 ng*hour/mL for TOSYMRA 10 mg, 4 mg SC injection, and 6 mg SC injection, respectively. Pharmacokinetic studies comparing a single dose of 10 mg TOSYMRA to 20 mg IMITREX demonstrated that TOSYMRA was more rapidly absorbed, with a C_max_ of 63.9 and 21.4 ng/mL and an AUC_0–2hr_ of 48.4 and 24.7 ng*hour/mL for TOSYMRA and IMITREX, respectively [76]. One multicenter, randomized, two-period, double-blind, placebo-controlled efficacy, safety, and tolerability study, in which patients were instructed to treat a single migraine episode using a single dose, demonstrated that a significantly greater percentage of patients achieved headache relief (defined as no or mild pain) at 2 h with 10 mg TOSYMRA compared to placebo (83.3% versus 55.0%, *p* = 0.005). Application site pain (2–2.7%) and dysgeusia (2–8.1%) were the most commonly reported AEs [78]. An open-label, long-term safety and tolerability study performed in patients who experienced two to six migraine headaches per month revealed that TOSYMRA was well tolerated when used over 6 months. A total of 52.7% of patients reported medication-related events. The most common AEs reported by ≥2% of patients were application site pain (30.5%), dysgeusia (21%), application site reaction (5.4%), upper respiratory infection (10.8%), nasopharyngitis (7.2%), and sinusitis (6.6%). Additionally, most patients (58%) reported the use of rescue medication at least once during the 6-month study [77]. No consistency of response data or long-term efficacy data were found in published literature.

### 3.2. Comparator Studies between Nasal Routes of Delivery and Oral Tablets

Studies have suggested that nasal delivery of some triptans provides more rapid onset with greater efficacy compared with oral triptan tablets [66,71]. The COMPASS study was a randomized, active-comparator, double-dummy, crossover, multi-attack study that compared the efficacy, tolerability, and safety of breath-powered nasal delivery containing a low dose (22 mg, AVP-825 (ONZETRA Xsail)) of sumatriptan versus oral delivery (100 mg) of sumatriptan. Results showed that ONZETRA plus placebo pill (ONZETRA) resulted in a significantly greater reduction in migraine pain intensity compared to 100 mg oral sumatriptan plus placebo nasal product (oral sumatriptan) in the first 30 min post-dose (least squares (LS) mean for summed pain intensity differences (SPID) = 10.8 versus 7.4, adjusted mean difference 3.4, *p* < 0.001). Greater rates of pain relief occurred with ONZETRA compared to oral sumatriptan at each time point measured: 15 min (27.9% versus 20.9%, *p* = 0.007), 30 min (53.8% versus 38.7%, *p* < 0.001), 45 min (65.0% versus 53.9%, *p* < 0.001), 60 min (72.1% versus 62.6%, *p* < 0.001), and 90 min (77.4% versus 72.0%, *p* = 0.03). Additionally, greater rates of pain freedom occurred with ONZETRA compared to oral sumatriptan at 15 min (7.2% versus 3.7%, *p* = 0.008), 30 min (18.2% versus 10.8%, *p* < 0.001), 45 min (31.0% versus 21.3%, *p* < 0.001), 60 min (41.2% versus 32.9%, *p* = 0.002), and 90 min (52.8% versus 44.9%, *p* = 0.006). It was postulated that early rates of pain relief may reflect quick systemic absorption of the sumatriptan powder delivered to the highly absorptive upper nasal cavity via the breath-powered device. Rates of pain relief and freedom at 2 h and sustained pain freedom from 2 to 48 h for ONZETRA were comparable to oral sumatriptan. This study also demonstrated that ONZETRA was well tolerated, as no serious AEs occurred, and most AEs were mild in severity. The most commonly reported AEs were abnormal product taste (26% ONZETRA versus 3.9% 100 mg oral sumatriptan) and nasal discomfort (15.5% ONZETRA versus 1.3% 100 mg oral sumatriptan). Additionally, ONZETRA displayed significantly fewer triptan-related AEs, such as a warm or burning sensation, feeling of heaviness, pressure, tightness, or numbness, compared to 100 mg oral sumatriptan (2% versus 5%, *p* = 0.02) [71].

A randomized, double-blind, placebo-controlled, multicenter, dose-ranging study compared the efficacy and tolerability of fixed doses of zolmitriptan administered via a nasal spray (ZOMIG) to both placebo and oral zolmitriptan tablets. Results revealed that each dose of ZOMIG produced a greater 2-hour headache response (no or mild pain; 70.3%, 58.6%, 54.8%, and 41.5% for ZOMIG 5, 2.5, 1, and 0.5 mg, respectively) compared with 30.6% for placebo (all *p* < 0.001 versus placebo). The 2-hour headache response rate for 5 mg ZOMIG was significantly higher than that of the 2.5 mg oral zolmitriptan tablet (70.3% versus 61.3%, *p* < 0.05). ZOMIG 5 mg provided a 2-hour headache response (11.1%) that was statistically superior to both placebo (5.4%) and the 2.5 mg oral zolmitriptan tablet (5.4%) at 15 min after administration (*p* < 0.05) [66].

### 3.3. Products in Development

#### 3.3.1. STS101

A nasal DHE powder delivered from a disposable nasal delivery device (STS101) is currently in development by Satsuma Pharmaceuticals (South San Francisco, CA, USA). A Phase 1, randomized, open-label, safety, tolerability, and comparative bioavailability study of STS101 demonstrated quick systemic absorption, attaining effective DHE plasma concentrations (>1000 pg/mL) within 10 min. Although the pharmacokinetics of STS101 demonstrated values that were 2.3-fold higher than those of MIGRANAL, it utilized a 6 mg dose that was 300% of the approved MIGRANAL 2 mg dose [89]. The results of topline data from the EMERGE trial, a Phase 3, multicenter, single-dose, randomized, double-blind, placebo-controlled, parallel-group efficacy study, showed that although numerical differences were in favor of STS101, the study did not demonstrate statistically significant differences between dosage strengths (4 and 6 mg) compared to placebo on coprimary endpoints of pain and most bothersome symptom freedom (among photophobia, phonophobia, or nausea) at 2 h post-administration [90].

#### 3.3.2. INP104

Impel NeuroPharma (Seattle, WA, USA) currently has a novel drug–device combination product in development (and submitted a new drug application (NDA) in November 2020) that targets delivery of liquid DHE mesylate to the upper nasal cavity using a Precision Olfactory Delivery (POD^®^) device (INP104). The POD technology has been developed to address the low bioavailability and variability in nasal administration seen with traditional nasal sprays. The drug delivery of INP104 to the upper nasal space takes advantage of the olfactory region’s abundant vascularity and avoids drug loss due to the drug dripping out of the nose or clearance to the nasopharynx, thereby increasing systemic availability [22,23,34,44,50,51]. A Phase 1, open-label, randomized, three-period, three-way crossover study evaluated the bioavailability of INP104. Healthy subjects received single doses of (A) INP104 1.45 mg, (B) IV DHE mesylate 1 mg, and (C) DHE mesylate nasal spray 2 mg (MIGRANAL) in one of six sequences with a 7-day washout between treatments. A 10 mg metoclopramide pretreatment was administered to all subjects [51]. Results revealed that exposure to DHE following administration of INP104 fell between that of IV DHE and MIGRANAL, with a four-fold increase in C_max_ and a three-fold increase in exposure (measured as AUC_0-inf_) compared to MIGRANAL, despite using an identical formulation, and <75% of the dose in the same healthy adult volunteers. INP104 produced a plasma DHE level that was comparable to that of IV DHE after only 30 min. The absolute bioavailability for INP104 was four times as much as that of MIGRANAL (58.9% versus 15.2%). Additionally, INP104 administration demonstrated less variability (coefficient of variation (CV%)) in C_max_ at 53.3% vs. 79.4% and in AUC_0-inf_ at 41.8% vs. 74.7% compared to MIGRANAL administration, suggesting more consistent drug delivery with the POD device [51]. INP104 was well tolerated, producing a tolerability profile comparable to that of MIGRANAL, but with less nausea than what is reported for MIGRANAL (10% in the MIGRANAL USPI) [51,62]. Importantly, drug leakage from the nose was only reported by 32% of INP104 users, compared to 77% of MIGRANAL users [51]. A Phase 3, interventional, open-label, single-group assignment study assessing the long-term safety and tolerability of chronic, intermittent use of INP104 over 24 weeks, with a treatment continuation to 52 weeks for a subset of users, was recently completed (STOP 301). The study revealed INP104 to be well tolerated over the course of a year [91], and detailed study results will be reported in an upcoming publication this year. Importantly, rates of nausea in patients who self-administered at least one dose of INP104 were low considering pretreatment with an antiemetic was not required. Of the total doses of INP104 administered over 52 weeks, INP104-related nausea was reported at a rate of less than 1%.

## 4. Does Nasal Delivery Address Patient Needs?

A 2017 study revealed that 95% of patients have at least one unmet need from their acute medication used to treat migraine. Many patients (74.1%) reported unmet needs associated with inadequate treatment response. Specifically, inadequate pain freedom at 2 h (48.1%) and headache recurrence within 24 h of initial relief (38%) were the two most common unmet needs associated with treatment. Patients (89.5%) also reported attack-related unmet needs, including the lack of rapid onset (65.3%) and headache-related disability (55.6%) [92]. A recent survey that assessed which medication attributes were of most interest to patients revealed that an ideal acute medication would be fast-acting (15–30 min) and long-lasting (12–24 h), would provide complete or near complete pain relief, could be taken at any time during the migraine, and would have few or no side effects. Patients also reported that they were willing to accept minor side effects as a trade-off for increased speed and efficacy [93]. These results suggest that the current treatment approach for many migraine patients is suboptimal and may explain treatment dissatisfaction [92,93]. Although oral medications are often effective, their onset of action may be slow due to gastric stasis that may be exacerbated in a migraine attack and the rate of subsequent absorption from the small intestine, and these delays may be worse for patients with nausea or vomiting. Such symptoms, which can be bothersome, even the most bothersome, may discourage patients from taking oral medication, and once vomiting has occurred after oral medication has been taken, may cause anxiety about whether to take a repeat dose. Administration of drugs via nasal delivery may overcome some of the limitations of oral administration, providing rapid absorption and resulting in swift onset of action [11,19,66].

Nasal delivery can provide an attractive avenue to achieve the consensus goals of rapid and consistent freedom from pain (Table 2). Rates of early pain relief and pain freedom favor nasal delivery over oral delivery for some triptans, with nasal delivery offering relief in as little as 15 min post-dose [71,72,73]. Additionally, nasal delivery can result in less headache-related disability and migraine-associated symptoms compared to oral delivery [71]. Nasal delivery offers consistency in headache response and lasting, durable relief from pain [66,71,73]. Finally, nasal delivery provides patients with the power to decide when and where to take treatment from easy-to-use, portable devices that may allow them to take control of their disease [22,23].

## 5. Conclusions

Nasal delivery is a well-established route of drug administration. However, the majority of nasal sprays for the acute treatment of migraine target the lower nasal space, where absorption is limited due to quick elimination from nasal drip or clearance to the nasopharynx and rapid mucociliary clearance. Upper nasal delivery provides well-tolerated, rapid, and efficient drug absorption, and improved bioavailability compared to lower nasal delivery, ensuring quick and durable migraine relief. Delivery of drugs to the highly vascularized and absorptive upper nasal space may be an optimal route for migraine therapy and, although few products targeting the upper nasal space exist, this technology expands the possibilities of nasal drug delivery with an easy-to-use, portable device.

## Figures and Tables

**Table 1:** Approved nasal products for the acute treatment of migraine.

Product	Initial US Approval Date	Key Product Details	Dosage	BAV	Key Efficacy Details	Key Safety Details
IMITREX^®^ (sumatriptan); GlaxoSmithKline, Research Triangle Park, NC, USA [29,60,61]	1997	- Liquid formulation delivered via traditional nasal spray - Indicated for the acute treatment of migraine with or without aura	5, 10, or 20 mg	17% relative to SC	- Eight randomized, double-blind, placebo-controlled trials (5 used recommended dosing/ marketed formulation) - Results reported for a single attack - Significantly greater percentage of patients achieved headache relief at 2 h post-treatment with 10 (*p* ˂ 0.05) or 20 mg (*p* ˂ 0.05) IMITREX vs. placebo in 4/5 studies	- Most commonly reported AEs in controlled studies were burning sensation, disorder/discomfort of nasal cavity/sinuses, throat discomfort, nausea, vomiting, bad/unusual taste, and dizziness/vertigo
MIGRANAL^®^ (dihydroergotamine mesylate); Bausch Health Companies Inc. or its affiliates, Bridgewater, NJ, USA [23,51,62,63]	1997	- Liquid formulation delivered to the lower nasal space - Indicated for the acute treatment of migraine with or without aura	2 mg	32% relative to IV	- Four randomized, double-blind, placebo-controlled, single-attack studies - Significantly greater percentage of patients achieved headache relief with 2 mg MIGRANAL vs. placebo at 2 h post-treatment in 1/4 studies (*p* < 0.001) and at 4 h post-treatment in 3/4 studies (*p* < 0.01)	- Most commonly reported AEs in controlled studies were rhinitis, altered sense of taste, application site reactions, dizziness, nausea, and vomiting - Black box label reports serious and/or life-threatening peripheral ischemia has been associated with coadministration of DHE and CYP 3A4 inhibitors
ZOMIG^®^ (zolmitriptan); Amneal Pharmaceuticals, Bridgewater, NJ, USA [33,64,65,66,67,68]	2003	- Liquid formulation delivered to the nasopharynx and lower nasal space - Indicated for the acute treatment of migraine with or without aura	2.5 or 5 mg	102% relative to oral	- One multi-attack trial for adults - One single-attack trial for adolescents (12–17 years) - Both randomized, double-blind, placebo-controlled, dose-ranging trials - Significantly greater percentage of patients achieved headache relief at 2 h with 2.5 or 5 mg ZOMIG vs. placebo in adults (*p* < 0.001) and adolescents (*p* < 0.05) - A randomized, double-blind, parallel-group, multicenter study designed as a two-phase, crossover trial assessed long-term tolerability, efficacy, and consistency of response of ZOMIG over 1 year	- Most commonly reported AEs in controlled studies were unusual taste, paresthesia, dizziness, and hyperesthesia in adults and unusual taste in adolescents - Long-term safety study revealed 5 mg ZOMIG was well tolerated over the course of 6 months, with unusual taste and paresthesia being the most frequently occurring AEs
ONZETRA^®^ Xsail^®^ (sumatriptan); Currax Pharmaceuticals, Morristown, NJ, USA [69,70,71,72,73]	2016	- Nasal powder delivered via breath to the upper nasal space - Indicated for the acute treatment of migraine with or without aura	22 mg	19% relative to SC	- One multicenter, randomized, double-blind, placebo-controlled, single-attack study - Significantly greater percentage of patients achieved headache relief at 2 h with 22 mg ONZETRA Xsail vs. placebo (*p* < 0.05) - Consistency of response was assessed in a randomized, multicenter, double-dummy, crossover, multi-attack, comparative efficacy study	- Most commonly reported AEs in the controlled study were abnormal taste, nasal discomfort, rhinorrhea, and rhinitis
TOSYMRA™ (sumatriptan); Upsher-Smith Laboratories, Maple Grove, MN, USA [74,75,76,77,78]	2019	- Liquid formulation containing a permeation-enhancing excipient (DDM, Intraveil^®^) - Indicated for the acute treatment of migraine with or without aura	10 mg	- 87% relative to 4 mg SC - 58% relative to 6 mg SC	- One multicenter, randomized, 2-period, double-blind, placebo-controlled, single-attack study - Significantly greater percentage of patients achieved headache relief at 2 h with 10 mg TOSYMRA vs. placebo (*p* = 0.005)	- Most commonly reported AEs in controlled studies were application site reactions and dysgeusia - The most common AEs reported over 6 months from the open-label study were application site pain, dysgeusia, application site reaction, upper respiratory infection, nasopharyngitis, and sinusitis

Note: This table is not a direct comparison. AE = adverse event; BAV = bioavailability; CYP = cytochrome P450; DDM = 0.2% 1-O-*n*-Dodecyl-β-D-maltopyranoside; DHE = dihydroergotamine mesylate; IV = intravenous; SC = subcutaneous; US = United States.

**Table 2:** How nasal delivery of acute treatments for migraine addresses attributes important to patients.

Attributes Desired by Patients	Upper Nasal Space Delivery	Traditional Nasal Delivery
Speed of onset—headache relief in <30 min [93,94,95]	- Relief reported at 15 min [72,73,85]	- Relief reported at 15–120 min [29,62,66]
Provides complete or near complete pain relief [94,95]	- Pain freedom reported at 30 min to 2 h post-dose - Sustained pain freedom through 48 h post-dose [71,72]	- Pain freedom/relief reported at 30 min to 4 h post-dose [29,62,66]
Few or minor side effects [94]	- Fewer AEs than oral delivery [71] - Commonly reported AEs include abnormal product taste, nasal discomfort, rhinorrhea, and rhinitis [71,72]	- May have high incidence of AEs local to the nasopharynx (i.e., intranasal paresthesia and unusual taste), with other AEs occurring at slightly higher rates overall compared to oral delivery [66] - Commonly reported AEs include nausea and/or vomiting, unusual taste, paresthesia, application site reactions, rhinitis, dysgeusia, throat irritation, intranasal paresthesia, hyperesthesia, dizziness, somnolence, and pharyngitis [29,62,64,66]
Relief from headache-associated symptoms [95]	- MBS relief as early as 10–15 min post-dose [71]	- MBS relief at 2–4 h post-dose [29,62,66]
Ability to carry on with the day [94]	- Majority of patients reported return to normal activities faster than their previous prescription [96]	- Allows for continuation of normal activities by 2 h in most patients [66,84]

Note: This table is not a direct comparison. AE = adverse event; MBS = most bothersome symptom; h = hour.

## Data Availability

Not applicable for this review of published work.

## Abbreviations

AE = adverse event; API = active pharmaceutical ingredient; AUC_0-inf_ = area under the drug concentration–time curve from time 0 to infinity; AUC_tmax_ = area under the serum concentration–time curve from zero to t_max_; BAV = bioavailability; C_max_ = maximum observed plasma concentration; CV% = coefficient of variation; CYP = cytochrome P450; DDM = *n*-Dodecyl-β-D-maltopyranoside; DHE = dihydroergotamine mesylate; FSS = full safety set; GI = gastrointestinal; IM = intramuscular; IV = intravenous; LM = least squares mean; NDA = new drug application; NF = National Formulary; POD = Precision Olfactory Delivery; SC= subcutaneous; SPID = summed pain intensity differences; TEAE = treatment-emergent adverse event; t_max_ = time to maximum plasma concentration; UAS = unilateral cranial autonomic symptoms; US = United States; USP = United States Pharmacopeia; USPI = United States Prescribing Information.
